# Mitochondrial Damage in the Trabecular Meshwork Occurs Only in Primary Open-Angle Glaucoma and in Pseudoexfoliative Glaucoma

**DOI:** 10.1371/journal.pone.0014567

**Published:** 2011-01-20

**Authors:** Alberto Izzotti, Mariagrazia Longobardi, Cristina Cartiglia, Sergio Claudio Saccà

**Affiliations:** 1 Department of Health Sciences, University of Genoa, Genoa, Italy; 2 Department of Head/Neck Pathologies, Ophthalmology Unit, St. Martino Hospital, Genoa, Italy; University of Florida, United States of America

## Abstract

**Background:**

Open-angle glaucoma appears to be induced by the malfunction of the trabecular meshwork cells due to injury induced by oxidative damage and mitochondrial impairment. Here, we report that, in fact, we have detected mitochondrial damage only in primary open-angle glaucoma and pseudo-exfoliation glaucoma, among several glaucoma types compared.

**Methodology/Principal Findings:**

Mitochondrial damage was evaluated by analyzing the common mitochondrial DNA deletion by real-time PCR in trabecular meshwork specimens collected at surgery from glaucomatous patients and controls. Glaucomatous patients included 38 patients affected by various glaucoma types: primary open-angle, pigmented, juvenile, congenital, pseudoexfoliative, acute, neovascular, and chronic closed-angle glaucoma. As control samples, we used 16 specimens collected from glaucoma-free corneal donors. Only primary open-angle glaucoma (3.0-fold) and pseudoexfoliative glaucoma (6.3-fold) showed significant increases in the amount of mitochondrial DNA deletion. In all other cases, deletion was similar to controls.

**Conclusions/Significance:**

Despite the fact that the trabecular meshwork is the most important tissue in the physiopathology of aqueous humor outflow in all glaucoma types, the present study provides new information regarding basic physiopathology of this tissue: only in primary open-angle and pseudoexfoliative glaucomas oxidative damage arising from mitochondrial failure play a role in the functional decay of trabecular meshwork.

## Introduction

Glaucoma is a neurodegenerative eye disease, the affected regions of which are (a) the retina, particularly the optic nerve; (b) the central nervous system, especially the lateral geniculate nucleus of the brain [1]; and (c) the eye anterior chamber, specifically the trabecular meshwork (TM). TM is crucial in glaucoma pathogenesis because its malfunction causes intraocular pressure (IOP) increases, as occur during open-angle glaucoma [2]. Indeed, TM cellularity, in particular the number of endothelial cells, plays a fundamental role in regulating the passage of fluids from the anterior chamber (AC) to Schlemm's canal [3]. Moreover, TM endothelial cells regulate the permeability of endothelial cells in Schlemm's canal, giving them a major role in the regulation of aqueous outflow [4]. The number of TM endothelial cells decreases with age [5]. During a patient's lifetime, TM cells are constantly exposed by free radicals in the AC, where a precarious balance between antioxidant defenses and oxidative free radicals exists [6]. Free radicals and reactive oxygen species are able to affect the cellularity of the human TM (HTM). These findings suggest that an intraocular pressure increase, which characterizes most glaucomas, is related to oxidative and degenerative processes affecting the HTM and, more specifically, its endothelial cells [7]. Furthermore, HTM is more sensitive to oxidative stress than other tissues around the AC [8]. Indeed, oxidative damage caused by reactive oxygen species (ROS) is greater in primary open-angle glaucoma (POAG) and pseudoexfoliative glaucoma (PEXG) patients than in healthy subjects [9]. TM endothelial cells secrete a number of factors, such as enzymes and cytokines, which modulate the functions of the cells and the extracellular matrix of the conventional aqueous outflow pathway [10] Damage to TM endothelial cells causes malfunction in the homeostasis of the outflow of the aqueous humor. The exact molecular mechanism underlying TM cell loss in glaucoma patients is still unclear. Indeed, chronic oxidative stress leads to the endogenous production of ROS by mitochondria in TM cells, thereby increasing the level of oxidative damage in the tissue [11]. In glaucomatous patients, a spectrum of mitochondrial abnormalities involving oxidative stress and implying mitochondrial dysfunction has been found [12]. The composition of aqueous humor proteins changes dramatically [13], and antioxidant proteins undergo down-regulation, in glaucoma; this leads to increased concentrations of nitric oxide synthase 2 and other proteins that, in physiological conditions, are segregated inside the functional mitochondria of TM cells [14]. Mitochondria are endowed with a specific DNA of maternal origin, mitochondrial DNA (mtDNA). This encodes only 13 structural proteins, all of which are involved in the respiratory chain. Most mitochondrial proteins are encoded by nuclear DNA. A complex interaction between the two genomes is therefore at the basis of mitochondrial protein synthesis and of mtDNA replication [15]. Mitochondria produce up to 90% of the cellular energy and also play a critical role in mediating cell death through the apoptotic pathway [16]. Damage accruing to the mitochondrial genome is associated with increased cellular stress and organelle dysfunction [17]. He and colleagues showed that TM cells of patients with POAG undergo ATP diminution, as their functionality is endangered by an intrinsic mitochondrial complex I defect, causing a respiratory chain deficit in these cells [18]. Recently, we reported that in TM of POAG patients, as compared with control subjects, mtDNA deletion is dramatically increased and the ratio between mtDNA and nuclear DNA is reduced. The amount of nuclear DNA in relation to wet tissue weight is also decreased [14]. These findings provide evidence that mitochondrial damage is severe in the TM of POAG patients.

Besides POAG, many other glaucoma types exist, including pigmented (PG), juvenile (JG), congenital (CG), acute closed-angle (ACG), neovascular (NVG), and chronic closed-angle glaucoma (CCAG), each one characterized by different and specific pathogenic mechanisms. The aim of the present paper is to establish whether there are biomolecular differences between mtDNA deletions in POAG and those found in other glaucoma types.

## Results

**Figure 1:**
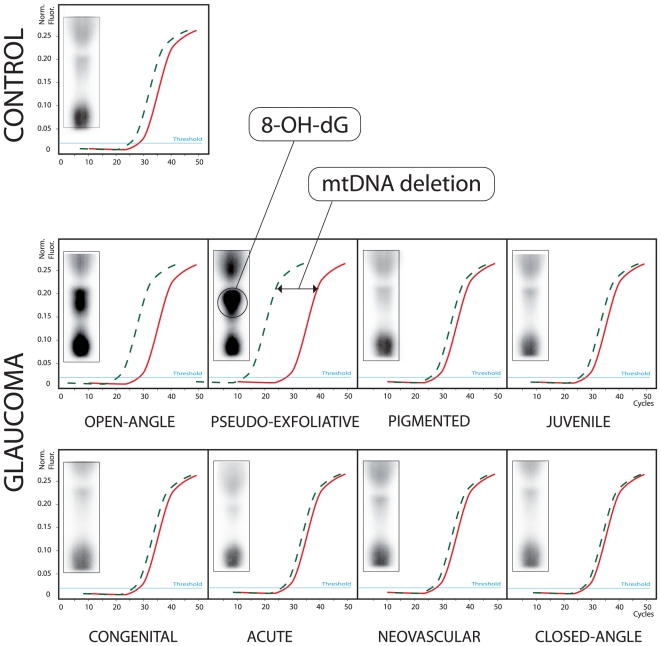
Normalized QPCR evaluation of the common 4977-bp mtDNA deletion in TM of patients with different glaucoma types. The horizontal axis shows the number of QPCR cycles, the vertical axis the amount of amplified DNA as inferred from the intensity of the fluorescence signal. The continuous curve indicates the normalized quantity of total mtDNA, the dashed curve indicates the relative amount of 4977 bp–deleted mtDNA. Samples in which the dashed curve intercepts the threshold horizontal line at lower QPCR cycles (left side) have a greater amount of 4977 bp–deleted mtDNA. For each sample, the box on the left shows oxidative nuclear DNA damage (8-oxo-dG) as detected by ^32^P-postlabeling.

The extent of mitochondrial DNA deletion in various glaucoma types as detected by QPCR is reported in Figure 1. These findings are paralleled by the amount of oxidative nuclear DNA damage (8-hydroxy-2′-deoxyguanosine) of the same samples by ^32^P-postlabeling (Figure 1).

Furthermore, a variety of parameters reflecting the occurrence of molecular alterations in mitochondria were considered, including (a) %mtDNA deletion/mtDNA, which is the amount of deleted mtDNA compared to total mtDNA and thus directly expresses the extent of the 4977 mtDNA common lesion; (b) total mtDNA/mg wet tissue, which represents the amount of mitochondria in the analyzed TM biopsy; (c) total nDNA/mg wet tissue, which represents the number of cells in the analyzed TM biopsy. As reported in Table 1, only POAG and PEXG showed a high level of mitochondrial deletion as compared to controls. The mtDNA 4977 deletion values observed in PEXG were more than double those detected in POAG.

**Table 1:** Mitochondrial DNA damage detected in TM of various glaucoma types.

Disease status	Age (years)	mtDNA deletion amount (%mtDNA deletion/mtDNA)	Mitochondrial number (mtDNA/mg wet tissue)	TM cell number (nDNA/mg wet tissue)	Oxidative nDNA damage (8-oxo-dG/105 nucleotides)
ControlsN = 16	65±12	12.9±5.1	18.7±1.6	9.1±1.6	2.3±2.5
**POAG**n = 16	71±9	38.7±21.8*	2.9±0.5*	0.5±0.1*	8.0±4.2*
**PEXG**n = 5	72±4	82.1±39.0*	0.1±0.0*	0.1±0.1*	9.8±3.7*
**PG**n = 2	36±14	5.0±4.1	3.5±3.2	0.7±0.5*	NT
**JG**n = 4	43±2	2.7±1.3	0.7±0.6*	0.1±0.1*	3.0±2.8
**CG**n = 4	21±10	0.3±0.1	0.9±0.4*	0.2±0.1*	1.5±0.4
**ACG**n = 3	60±2	0.9±0.8	4.1±2.4*	0.9±0.7*	2.8±1.0
**NvG**n = 2	70±4	0.1±0.0	4.7±0.7*	0.7±0.3*	3.2±1.2
**CCAG**n = 2	64±2	0.4±0.3	1.8±1.5*	3.3±3.1*	2.1±0.4

*P<0.05 compared to controls.

In all glaucoma types other than POAG and PEXG, the amount of mitochondrial deletion did not significantly vary from that observed in control TM samples (Table 1).

Similar findings were obtained for oxidative damage to nuclear DNA, which significantly increased only in POAG and PEXG and not in other glaucoma types.

TM cellularity, expressed as total nDNA/mg wet tissue, was dramatically decreased in all glaucoma types, indicating that cell loss in TM is a non-specific event occurring in many glaucoma types. Similarly, the total number of mitochondria in TM decreased in all glaucoma types, a finding related to the cell loss observed. Conversely, the number of mitochondria per cell (mtDNA/nDNA ratio) was significantly decreased only in POAG and PEXG (Table 1).

A non-significant trend towards a lower level of mtDNA deletion in some glaucoma types (juvenile, congenital,) than in controls was observed, probably due to the younger age of these patients as compared to controls.

A significant correlation between the amount of mtDNA deletion and the level of oxidative DNA damage detected in different glaucoma types was observed (*r* = +0.942, *P*<0.001). This finding indicates that mitochondrial damage is tightly correlated with the development of oxidative damage in TM cells. A significantly inverse relationship was observed between the number of mitochondria per TM cell and oxidative damage (r = −0.780, P<0.05). This finding indicates that accumulation of oxidative damage inside TM cells results in severe mitochondrial loss.

Together, our results provide evidence of the strong connection existing in TM between oxidative stress and mitochondrial damage in degenerative forms of glaucoma (POAG and PEXG).

## Discussion

Our results indicate that mitochondrial damage and related oxidative stress specifically occur in POAG and PEXG and not in other glaucoma types.

A high level of mitochondrial alterations was observed, particularly in PEXG, which had the highest levels of mtDNA deletion, oxidative nDNA damage, and mitochondrial loss per cell. These results justify the clinical features of PEXG, which is more aggressive than POAG and typically displays higher intraocular pressure [19]; moreover, tonometric compensation is also more difficult in PEXG.

These results indicate that POAG and PEXG are characterized by specific mechanisms involving mitochondrial damage, oxidative stress, and mitochondrial loss, finally resulting in TM cell loss. Conversely, in other glaucoma types, TM cell loss is independent of mitochondrial damage and loss.

The observed specific role of mitochondria damage in the degenerative glaucomas (i.e., POAG and PEXG) is in line with the important function of this organelle. Human cells depend on ATP for growth, differentiation and responses to physiological and environmental stimuli. Mitochondria are cytoplasmic organelles, the main role of which is to synthesize ATP. They play crucial roles in other cell processes, the most important of which include development, calcium homeostasis, cell cycle regulation, thermogenesis, free radical production and apoptosis. The respiratory chain is composed of five multiheteromeric enzymatic complexes (I, II, III, IV and V) embedded in the inner membrane of mitochondria. The respiratory chain is under the control of two separate genetic systems, i.e. mtDNA and nDNA behaving differently in many respects [20]. The mitochondrial genome regularly replicates in postmitotic cells, about once per month. The mitochondria divide mainly in response to the energy needs of the cell, i.e., independently of the cell cycle [21]. The mitochondria continuously fuse with one another, and the cells may therefore contain both normal and possibly modified mitochondrial genes. After cell division, the proportions of mutant genomes differ in the individual descendant cells. The frequency and proportion of the deleted mtDNA in human tissues increase with age. mtDNA deletions occur more frequently and abundantly in high energy-demanding tissues during the aging process [22]. mtDNA is more vulnerable to oxidative stress, its mutation rate being about 10-fold higher than that of nDNA [23]. All tissues from adult subjects show the presence of mitochondrial DNA molecules with deletions [24], and the accumulation of mtDNA mutations in aging contributes significantly to the decline of mitochondrial energy production that characterizes the aging process in many tissues [25]–[26].

In mitochondria, the production of reactive oxygen species (ROS) increases, as do the by-products of aerobic metabolism in aging tissues. Indeed, the mitochondrial respiratory chain is one of the main sources of deleterious free radicals in the human organism. Under normal physiological conditions, a small fraction of the oxygen consumed by mitochondria is constantly converted to superoxide anions, hydrogen peroxide, hydroxyl radicals and other ROS [27]. The age-dependent decline in the capacity of mitochondria to dispose of these oxy-radicals renders mtDNA more vulnerable to mutations during the ageing process [28]. In this light, the mtDNA deletions in the human TM that accumulate during the aging process are the result of this reduced capability. Mitochondrial decay, as evaluated by cytochrome *c* oxidase analysis, is associated with apoptotic activation in TM cells in patients with POAG [18], in that oxidative stress causes activation of mitochondrial matrix caspase activity, which is secondary to cytochrome *c* release [28]. In addition to the endogenous formation and accumulation of mtDNA deletions in human tissues during the normal aging process, physiological and environmental factors such as light may also play important roles in eliciting the deposition of deleted mtDNA molecules in cells [29]. Such mutations are induced through a similar mechanism as the endogenous mutations, although the absolute extent of accumulation differs among tissues [30]. Our results show that the generalized relative abundance of particular mtDNA mutations in specific tissues is associated with specific TM pathologies, i.e., PEXG and POAG.

Exfoliation syndrome, associated with PEXG, is an age-related disorder of the extracellular matrix characterized by the production and progressive accumulation of a fibrillar material not only in ocular tissues but also in skin and connective tissue portions of various visceral organs. From a histopathological point of view, PEXG is different from POAG. Whereas POAG is characterized by increased juxtacanalicular plaque material and decreased TM cellularity [5], in pseudoexfoliative glaucoma, both plaque material and cellularity are unchanged compared with normal eyes; the characteristic exfoliation material is deposited instead [31]. Although these histopathological differences suggest completely different pathogenetic mechanisms [32], our results show that POAG and PEXG share one mechanism in common. They both produce a decrease in TM cellularity through mitochondrial damage and apoptosis, which is stimulated by the ROS circulating in the AC and produced in TM by damaged mitochondria. Oxidative DNA damage in pseudoexfoliative patients is greater than that which occurs in POAG patients in the TM region. However, the accumulation of locally produced pseudoexfoliative material in the juxtacanalicular tissue, and the consequent dysfunction of endothelial cells and disorganization of juxtacanalicular tissue and Schlemm's canal, are causative factors in the development of a special type of secondary open-angle glaucoma in exfoliation syndrome [31]. Decreased cellularity of the trabecular meshwork appears to be a particular characteristic of POAG, but other authors reported that it does not seem to play a role in the pathogenesis of PEX glaucoma [31].

Interestingly, other types of glaucoma have not shown signs of mitochondrial deletion increases. In neovascular glaucoma, the TM is damaged by neo-vessels that invade the tissue, causing its malfunctioning and increasing IOP. Indeed, if neovascularization regresses, a rapid IOP decrease results [33]. Both chronic and acute closed-angle glaucomas present structural similarities: pupil-blocking force, status of angle synechia closure, and shallow depth of the anterior chamber; however, the former is asymptomatic, whereas the latter is symptomatic. In any case, both glaucomas share a final pathway involving the formation of irreversible synechial adhesion between the peripheral iris and the TM [34]. In closed-angle glaucomas, the TM is obstructed by the iris, which prevents TM cells from functioning, thereby impeding the normal flow of aqueous humor across this region. The duration of contact closure necessary to cause synechial closure appears to vary greatly from patient to patient. If the obstacle to outflow is promptly removed, IOP may return to normal values. However, prolonged contact between the iris or iris/lens and the TM may lead to permanent damage to TM functionality and produce a permanent IOP increase.

In congenital and juvenile glaucoma, there are several pathogenic factors that come into play, spanning from arrested development of the anterior chamber angle structures, which is characterized by incomplete maturation of the TM and Schlemm's canal [35], to mesodermal dysgenesis of the iris and cornea characterized by abnormalities of the anterior chamber cleavage [36] and from congenital hereditary endothelial dystrophy [37] to arginase deficit [38]. The increased resistance to aqueous humor outflow in congenital glaucoma may stem from abnormalities of the TM and the internal wall of Schlemm's canal [39]. It is possible that both congenital and juvenile glaucoma occur because of developmental immaturity of the TM in anterior chamber angle tissues [40]. Ultrastructural examination of TM shows that the inner cribriform area is greatly thickened and the amount of sheath-derived plaque material is increased in the subendothelial layer adjacent to Schlemm's canal [41]; there are no vacuoles in the endothelium of Schlemm's canal, and there is a broad layer of collagen and amorphous material in the juxtacanalicular connective tissue [42]. The class and distribution of proteoglycans in the goniodsygenetic trabecular tissues are virtually the same as in normal tissues [43], and there are no significant differences between the morphological picture of primary congenital glaucoma and that found in Axenfeld and Rieger anomalies [44]. Accordingly, the pathogenesis of all these glaucoma types does not require the occurrence of mitochondrial damage, as demonstrated by the data reported here.

The lack of mitochondrial deletion was also confirmed in the cases of pigmentary glaucoma. In this situation, the development of the chronic glaucomatous condition cannot be directly attributed to pigment accumulation in the juxtacanalicular tissue [45]. However, many TM alterations have been described: loss of trabecular cells, fusion of trabecular lamellae and collapse of intertrabecular spaces, increase in extracellular material, and obliteration of the canal have been found to varying degrees around the circumference of eyes with pigment dispersion syndrome and elevated intraocular pressure and in pigmentary glaucoma. These probably all contribute to the development of increased intraocular pressure in pigmentary glaucoma [46]. Macrophages are the main cell type responsible for TM clearance of pigment and debris [47]. It is possible that, on a genetic basis, they play a central role in the pathophysiology of pigmentary glaucoma.

In conclusion, our findings demonstrate that in all types of glaucoma in which IOP is high, TM malfunction resulting from cellularity decrease is the key to the development of the disease. However, only in primary open angle and pseudo-exfoliation glaucoma was mitochondrial damage detected, as the outflow dysfunction in the other glaucomas studied may have a different underlying basis.

## Materials and Methods

### Patient recruitment and sample collection

Between January 2007 and July 2009, 38 consecutive glaucoma patients (21 women, 17 men) aged 55–82 years (70±7.15 years, mean ± SE) were enrolled. They included 16 cases of primary open-angle (POAG), 2 pigmented (PG), 4 juvenile (JG), 4 congenital (CG), 5 pseudoexfoliative (PEXG), 3 acute (ACG), 2 neovascular (NvG), and 2 chronic closed-angle (CCAG) glaucoma.

Various glaucoma diagnosis were performed on a clinical basis. Both NVG patients were suffering from systemic hypertension and both have had a thrombosis of central vein of the retina.

Glaucoma was diagnosed and classified on the basis of clinical characteristics (slit-lamp examination, gonioscopy, intraocular pressure (IOP) increase, pachymetry, perimetric test and optic nerve morphometric or nerve fiber layer analysis with GDx or HRT) according to the classification of Shields, Ritch and Krupin [48]. GDx-VCC software version 5.2.3 (Laser Diagnostic Technologies, San Diego, CA) was used to evaluate the parapapillary RNFL. Confocal scanning laser ophthalmoscopy was performed with the Heidelberg Retina Tomograph II and analyzed with HRT III software (Heidelberg Engineering GmbH, Heidelberg, Germany). Visual-field testing was performed with the Humphrey Field Analyzer 750 (Allergan Humphrey, San Leandro, CA). The presence of perimetric glaucomatous defects was assessed by computerized visual-field testing (Humphrey 30-2 computerized visual-field examination) and graded according to the criteria proposed by Caprioli [49]. A visual-field defect score was assigned according to Brusini and Tosoni [50]. The study adhered to the tenets of the Declaration of Helsinki and was approved by the Ethical Board of the Ophthalmologic Division. The glaucoma patients, after having provided written informed consent, underwent ocular filtration surgery for therapeutic purposes. Trabeculectomy specimens were collected at surgery from the 38 glaucomatous patients. For each patient, we considered only one eye with an established diagnosis of POAG showing failed tonometric compensation and worsening of perimetric and optic disc conditions. All patients were treated with maximal therapy, including prostaglandin F2 analogs (92.1% [35 patients]), systemic carbonic anhydrase inhibitors (86.8% [33 patients]), topical beta-blockers (84.2% [32 patients]), topical carbonic anhydrase inhibitors (84.2% [32 patients]), α_2_-agonists (76.3% [29 patients]), and pilocarpine (28.9% [11patients]). Despite using the maximal tolerated therapy, all patients showed elevated IOP on entry to the hospital department (min 25 mmHg; max 41 mmHg; mean 31.3±4.62). Patients underwent ocular filtration surgery for therapeutic purposes at the Ophthalmology Unit of S. Martino Hospital in Genoa from 2007 to 2009. Samples were collected by standard surgical trabeculectomy as previously described [5]–[9]. Inclusion criteria were the presence of glaucoma with no tonometric compensation and the absence of any other ocular, systemic or neurological diseases other than optic nerve damage.

As control samples, we used trabeculectomy and iris specimens collected from 16 subjects aged 66.1±1.27 years who were gender-matched with patients. These samples were provided by the Melvin Jones Eye Bank in Genoa from 2007 to 2009. The control samples had been obtained immediately after death from glaucoma-free subjects undergoing eye removal for cornea transplantation, as previously described [9]. Every effort was made to ensure that the sampling times in donors were the same as in the patients studied. Control samples were taken no later than 1 hour after death, thus ensuring cell viability, which is obviously very important in cornea transplantation. Samples were immersed in stabilizing buffers containing antioxidants and stored at −80°C until DNA purification, which was performed with proteinase K and solvent extraction in an oxygen-free atmosphere, as previously described [9].

Approval for this study was given by the Ethics Committee of the Ophthalmologic Unit of the Head/Neck Department of San Martino Hospital, Genoa, Italy, and all study patients provided written informed consent. The research followed the tenets of the Declaration of Helsinki.

### Detection of mitochondrial DNA damage

Mitochondrial DNA damage was evaluated in terms of the common 4977-bp deletion, which is a large mtDNA deletion involving the loss of almost one third of the mitochondrial genome.

To evaluate the frequency of the common 4977-bp deletion in mtDNA, we performed two real-time QPCR reactions for each sample, one aimed at detecting the amount of total mtDNA (TMDQ) and one to quantify the deleted mtDNA (DMDQ). The TMDQ evaluation was carried out by using two primers (tot mt S: CCATCTTTGCAGGCACACTCATC and tot mt A: ATCCACCTCAACTGCCTGCTATG) flanking a sequence of mt DNA known not to be susceptible to deletion [51] and an *ad hoc*–designed FAM-labeled molecular beacon (mt tot: CGCGATCTCACGCAAGCAACCGCATCCATGATCGCG). The DMDQ evaluation was performed using two primers (del mt S: GGCCCGTATTTACCCTATAG and del mt A: GGTGAGAAGAATTATTCGAGTG) flanking the region susceptible to deletion and a second *ad hoc* FAM-labeled molecular beacon (mt del: CGCGATCCAGCCTAGCATTAGCAGGAATACCTTGATCGCG). Because of the short elongation time, only deleted mtDNA can efficiently give rise to an amplicon containing the target sequence for the mt del molecular beacon. DNA for TMDQ quantification was diluted 10-fold more than for DMDQ. The reaction conditions were 5 µl 10x PCR buffer, 0.4 µl 100 mM dNTP mix, 2 µl 50 mM MgCl_2_, 0.5 µl Platinum *Taq* DNA polymerase (Invitrogen Corporation, Carlsbad, CA), 37.1 µl sterile water, 1 µl 10 mM sense primer, 1 µl 10 mM anti-sense primer, 2 µl molecular beacon, and 1 µl DNA. The mixture was split into two 200-µl flat-cap transparent tubes.

The reactions were performed in a Rotorgene 3000 (Corbett Research, Mortlake NSW 2137, Australia) as follows: 94°C for 2 min, followed by 45 cycles at 94°C for 30 sec, room temperature for 30 sec, and 72°C for 30 sec. The FAM channel was acquired at the end of the annealing step. The annealing temperature for mt tot was 54°C and for mt del 51°C.

The del-mtDNA/tot-mtDNA ratio was assessed for each sample and normalized to the number of cells and to the quantitiy of nuclear DNA, as assessed by a further QPCR reaction quantifying the housekeeping gene GAPDH.

Primer sequences and PCR conditions were determined by using Beacon Designer 6.0 software (Premier Biosoft International, Palo Alto, CA). Result specificity was checked by analyzing the melting curves of PCR products.

The relative amount of total nuclear DNA in each sample was evaluated by quantifying the copies of GAPDH mRNA by means of quantitative real-time PCR (QPCR). This parameter was compared both to the number of mitochondrial DNA copies (nuclear/mitochondrial DNA ratio) and to the amount of wet tissue processed (DNA/g wet tissue). This last parameter was taken as an indicator of TM cellularity. The following primers and the molecular beacon specific for a 149-bp DNA sequence of human GAPDH were designed with Beacon Designer and purchased from TIB (Molbiol, Berlin, Germany): Sense TGTCTGCTTCTCTGCTGTAG, Anti-sense CTGCCTTCCTCACCTGATG, Probe 5′ – FAM – CGCGATCCATGTTCGTCATGGGTGTGAACCAGATCGCG – BHQ1.

The QPCR reaction was carried out in a rotating real-time thermocycler (Corbett Life Science, Sydney, Australia) in a mixture containing 0.1 µg DNA, 0.2 mM each of ATP, GTP, TTP and CTP, 1.5 mM MgCl_2_, 2 U Platinum *Taq* DNA polymerase, 0.2 µM primer Sense, 0.2 µM primer Anti-sense, 0.4 µm Probe and water to 50 µl. Polymerase was hot start–activated at 94°C for 2 min, and 40 amplification cycles of 94°C for 40 sec, 53°C for 30 sec and 72°C for 30 sec followed. The fluorescent signal was detected at every annealing step.

Relative quantification was performed by comparing the cycle threshold of every sample with that of a standard dilution of a DNA sample of known concentration.

### Detection of oxidative DNA damage to nuclear DNA

Oxidative DNA damage was analyzed in terms of 8-hydroxy-2′-deoxyguanosine (8-oxo-dG) by ^32^P-postlabeling as previously reported [9].

### Statistical analyses

Comparisons of quantitative variables among different groups were performed by ANOVA and Student's *t* test for unpaired data after checking the normality of the distribution by kurtosis skew analysis. The accepted level of significance in all cases was *P*<0.05. In the case of non-normal distribution of data, non-parametric Mann-Whitney U and Kruskal-Wallis tests were used. Correlations between continuous variables were tested by linear regression analysis. Differences in frequency distributions among nominal variables were tested by the chi-square test. All statistical analyses were performed with the Statview software (Abacus Concepts, Berkeley, CA).
